# Supplementary material for: The microbiome biomarkers of pregnant women’s vaginal area predict preterm prelabor rupture in Western China
Source: Front Cell Infect Microbiol. 2024 Oct 31;14:1471027. doi: 10.3389/fcimb.2024.1471027 (PMC11560878; doi:10.3389/fcimb.2024.1471027)
Supplement: Supplementary file 1 [file DataSheet1.zip › compare_1/Community/Structure_Barplot/species/species.taxon.Barplot.Legend.With.genus.pdf]

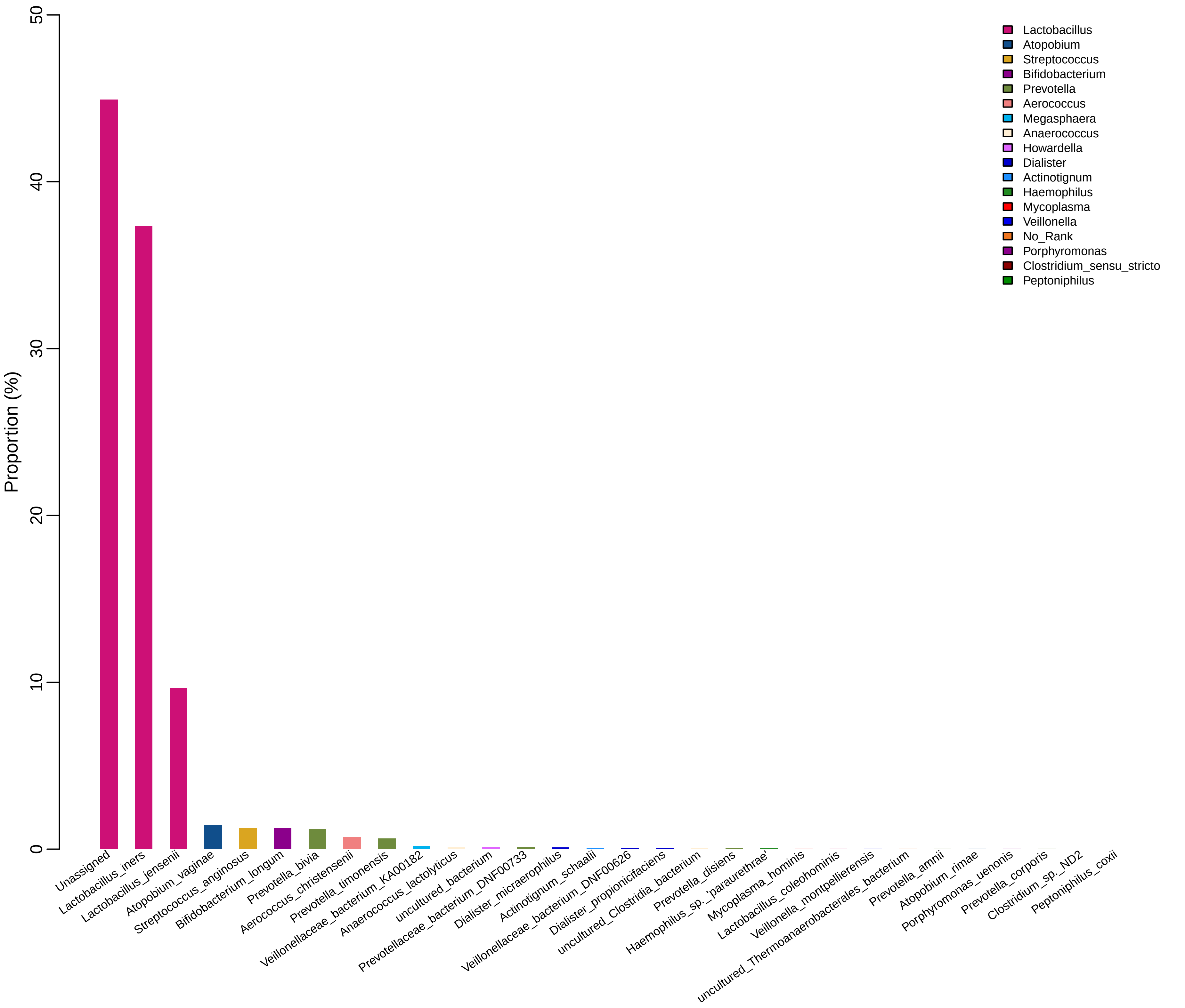

Proportion (%)
0
10
20
30
40
50
Lactobacillus
Atopobium
Streptococcus
Bifidobacterium
Prevotella
Aerococcus
Megasphaera
Anaerococcus
Howardella
Dialister
Actinotignum
Haemophilus
Mycoplasma
Veillonella
No_Rank
Porphyromonas
Clostridium_sensu_stricto
Peptoniphilus
Unassigned
Lactobacillus_iners
Lactobacillus_jensenii
Atopobium_vaginae
Streptococcus_anginosus
Bifidobacterium_longum
Prevotella_bivia
Aerococcus_christensenii
Prevotella_timonensis
Veillonellaceae_bacterium_KA00182
Anaerococcus_lactolyticus
uncultured_bacterium
Prevotellaceae_bacterium_DNF00733
Dialister_micraerophilus
Actinotignum_schaalii
Veillonellaceae_bacterium_DNF00626
Dialister_propionicifaciens
uncultured_Clostridia_bacterium
Prevotella_disiens
Haemophilus_sp._'parauretrhae'
Mycoplasma_hominis
Lactobacillus_coleohominis
Veillonella_montpellierensis
uncultured_Thermoanaerobacterales_bacterium
Prevotella_amnii
Atopobium_rimae
Porphyromonas_uenonis
Prevotella_corporis
Clostridium_sp._ND2
Peptoniphilus_coxii
